# Optimizing Ventana chromogenic dual in-situ hybridization for mucinous epithelial ovarian cancer

**DOI:** 10.1186/1756-0500-6-562

**Published:** 2013-12-28

**Authors:** Xinyun Li, Sung-Hock Chew, Wen-Yee Chay, Soo-Kim Lim-Tan, Liang-Kee Goh

**Affiliations:** 1 School of Biological Sciences, Nanyang Technological University, Singapore, Singapore; 2 Department of Pathology, KK Women’s and Children’s Hospital, Singapore, Singapore; 3 Department of Medical Oncology, National Cancer Centre, Singapore, Singapore; 4 Cancer & Stem Cell Biology, Duke-National University of Singapore, Singapore, Singapore; 5 Saw Swee Hock School of Public Health, National University of Singapore, Singapore, Singapore

**Keywords:** Dual in-situ hybridization, Fluorescence in-situ hybridization, Formalin-fixed paraffin-embedded tissue, Human epidermal growth factor 2, Mucinous epithelial ovarian cancer, Optimization protocols

## Abstract

**Background:**

Dual in-situ hybridization (DISH) assay is a relatively new assay for evaluating Human Epidermal Growth Factor Receptor 2 (HER2) genomic amplification. Optimization protocol for the assay is not yet well established, especially for archival tissues. Although there is a recommended nominal protocol, it is not suited for formalin-fixed and paraffin-embedded (FFPE) samples that were archived for long periods.

**Findings:**

In a study on local population of mucinous epithelial ovarian cancer, we developed a series of optimization protocols based on the age of samples to improve success of the DISH assay. A decision workflow was generated to facilitate individualization of further optimization protocols. The optimizations were evaluated on 92 whole tissue sections of FFPE mucinous ovarian tumors dating from 1990 to 2011. Overall, 79 samples were successfully assayed for DISH using the series of optimization protocols. We found samples older than 1 year required further optimization beyond the nominal protocol recommended. Thirteen samples were not further assayed after first DISH assay due to inadequately preserved nuclear morphology with no ISH signals throughout the tissue section.

**Conclusion:**

The study revealed age of samples and storage conditions were major factors in successful DISH assays. Samples that were ten years or less in age, and archived in-house were successfully optimized, whereas older samples, which were also archived off-site, have a higher frequency of unsuccessful optimizations. The study provides practical and important guidelines for the new DISH assay which can facilitate successful HER2 evaluation in ovarian cancers and possibly other cancers as well.

## Background

Fluorescence in-situ hybridization (FISH) assay has been the gold standard in ascertainment of HER2 gene amplification in breast and gastric cancers with well established and proven protocols [1]. On the other hand, dual in-situ hybridization (DISH) is a relatively new assay, approved by the FDA in July 2011, with yet-to-be established optimization protocols. Recent studies have shown both assays are comparable in ovarian cancers [2,3]. FISH technique requires time-sensitive interpretation, a need for fluorescent microscope, and is more costly. In comparison, DISH assay is based on light microscopy; is more user-friendly and the slides are archivable, hence proving to be a more favorable option.

There is increasing evidence of significant HER2 gene amplification in mucinous epithelial ovarian cancer (mEOC), which is of great interest due to the potential of targeted therapy such as Trastuzumab or Pertuzumab. Of all epithelial ovarian cancer (EOC), mEOC is the least studied due to their relative rarity. These cancers do not respond well to chemotherapy and are associated with poor prognosis [4,5]. Our previous study looking at copy number alterations across four main histotypes of EOC revealed mEOC harbored highest prevalence of HER2 amplification (28.6%) in EOC and is a potential driver gene for copy number alterations [6]. Several studies have also shown high prevalence of HER2 in mEOC using immunohistochemistry (IHC) and in-situ hybridization assays. Mayr et al. [7] and McAlpine et al. [8] used IHC and FISH to determine the prevalence of HER2 protein overexpression and gene amplification. They reported 16.7% and 18.2% of mEOC were HER2 positive in their western cohorts (n = 17 and 33 respectively). In Yan et al. study [3], IHC, FISH and DISH were employed on a small Singapore cohort of samples (n = 17) dated from 2000 to 2010, reporting HER2 in 35.3% of mEOC. A recent large western cohort study on 154 mEOC samples showed 18.8% of mEOC were HER2 positive [2].

In our study on HER2 in local patients using DISH [9], we developed a series of optimization protocols and a decision workflow for archived FFPE samples dating as far back as 22 years. The establishment of these protocols has facilitated our clinical research on ovarian cancers, increasing success of the assay, especially for samples more than 1 year old. We believe these protocols will add value to others who utilize or intend to utilize DISH for HER2 ascertainment in ovarian cancers, and possibly other cancers such as breast and gastric cancers where HER2 is prevalent. In ovarian cancers where HER2 inhibitor is not yet incorporated in the treatment strategy, there is growing momentum to utilize HER2 inhibitor in treatment of mucinous ovarian cancer [2]. Hence, the optimization protocols may also be useful in clinical application, where FFPE samples of current or recurrent cases need to be reviewed for HER2 status.

## Materials and methods

### Patient specimens

Mucinous EOC cases (n = 92) diagnosed at KK Women’s and Children’s Hospital (KKH), Singapore, between years 1990 and 2011 were included in this study. Details of the patient specimens have been reported in another study on HER2 and the clinicopathological factors that influence the disease [9]. Briefly, samples dated from year 2003 onwards were archived in KKH while those from years 1990 to 2002 were archived off-site (Table 1). The specimens were obtained from patients who underwent unilateral or bilateral salpingo oophorectomy and/or total hysterectomy, and were routinely fixed in neutral buffered formalin (NBF) for variable durations. The histological classification of mEOC was determined by gynaecological pathologists at the time of diagnosis. Tissue specimens were FFPE and 3 μm serial sections were used for Hematoxylin and Eosin (H&E) staining to confirm the presence of mEOC. The H&E slides were then reviewed by the gynecological pathologist (SHC) who was involved in the study and those which fulfilled the criteria of primary invasive mEOC were selected.

**Table 1:** Summary of the samples that were used in this study – the year of diagnosis, the number of samples in each year, and the archival site are indicated

**Year of diagnosis**	**Number of samples**	**Archival site**
2011	3	KKH
2010	5	KKH
2009	4	KKH
2008	11	KKH
2007	9	KKH
2006	5	KKH
2005	5	KKH
2004	2	KKH
2003	4	KKH
2002	1	Off-site
2001	5	Off-site
2000	3	Off-site
1998	5	Off-site
1997	5	Off-site
1996	9	Off-site
1995	4	Off-site
1994	4	Off-site
1993	1	Off-site
1992	3	Off-site
1991	3	Off-site
1990	1	Off-site

### Ethics

This study was approved by the institutional review boards (IRB) of the National Cancer Centre Singapore, KK Women's and Children's Hospital Singapore and Singapore General Hospital Singapore. IRB waiver of informed consent was approved as analyses were performed retrospectively on non-identifiable data (CIRB 2010/425/B).

### Dual in-situ hybridization

DISH was performed in an automated BenchMark ULTRA (Ventana Medical Systems, USA) slide stainer, using the INFORM HER2 Dual ISH DNA Probe Cocktail Assay (Ventana Medical Systems, USA) that allows detection of HER2 gene amplification by light microscopy.

A total of 92 mEOC samples were evaluated for HER2 gene amplification status. There are nine main steps to the automated DISH – (i) baking to ensure adhesion of tissue sections to the slide, (ii) deparaffinization to remove the paraffin for reagent penetration, (iii) pretreatment to open protein crosslinks and expose nucleic acids, (iv) denaturation of double stranded DNA to expose the DNA targets, (v) hybridization of probes to target genes, (vi) stringency washes to wash off excess probes, (vii) indirect detection of hybridization event, (viii) counterstaining to enhance visualization, and (ix) coverslipping to protect the tissue sections.

The DISH protocol has been previously described [10]. Briefly, tissue sections were deparaffinized and pretreated with Cell Conditioning 2 (CC2) at pH 6 at 86°C. Enzymatic digestion of proteins was performed with ISH protease 2 or 3 for variable length of time. The double-stranded DNA was denatured, allowing hybridization of labeled probes - dinitrophenyl (DNP)-labeled HER2 DNA probe to the HER2 gene and digoxigenin (DIG)-labeled Chromosome 17 centromere probe (CEN17) to the centromere of chromosome 17. A stringency wash was performed at 72°C using sodium citrate, sodium chloride (SSC 10×) to wash off unbound or weakly bound probes. The ultra*View* Silver ISH DNP and ultra*View* Red ISH DIG detection kits (Ventana Medical Systems, USA) were used for the detection of HER2 and CEN17 signals respectively. For detection of HER2 gene, the slides were first incubated with rabbit anti-DNP antibodies, followed by horse radish peroxidase (HRP)-conjugated goat anti-rabbit antibodies. Reaction of Silver ISH DNP Chromogens A, B and C with HRP will produce black signals which represent the HER2 gene. For detection of CEN17 signals, the slides were first incubated with mouse anti-DIG antibodies, followed by alkaline phosphatase (AP)-conjugated goat anti-mouse antibodies. The substrates for AP were Red ISH DIG pH enhancer, naphthol and Fast Red. The enzymatic reaction will result in red signals which represent the centromere of chromosome 17. Tissue sections were then counterstained in hematoxylin II and bluing reagent to enhance contrast. To minimize evaporation of aqueous reagents from the slides, liquid coverslip (LCS) was applied to the tissue sections by the stainer. Upon completion, slides were unloaded. Tissue sections were soaked in a detergent bath to wash off LCS, followed by soaking in a water bath to rinse off the detergent. Tissue sections were then baked in an oven at 55°C for 30 minutes to dry the tissue sections before coverslipping was done in Cytoseal 60 mounting media (Richard Allan Scientific, USA). The expected outcome from the slides was optimal staining, i.e. distinct nuclear morphology without obscuration of the red and black signals, and without non-specific background staining. This is to ensure specificity in the enumeration of the red and black signals within each nucleus. For slides without optimal staining, further optimizations protocols were needed.

### Development of optimization protocols

DISH is a newly established in-situ hybridization assay and the parameters in the nominal protocol (U1) were developed for samples that are freshly fixed in formalin and embedded in paraffin, and not entirely applicable for the older samples. Therefore, optimization of the protocol parameters had to be performed in older samples to promote optimal staining. In general, six parameters were explored during optimization: (i) incubation times of the three cycles of CC2, (ii) use of either ISH proteases 2 or 3, and their incubation times, (iii) temperature of stringency wash, (iv) incubation times of SISH and Red ISH multimers, (v) incubation times of silver and red chromogens, (vi) incubation times of Hematoxylin II and bluing reagent. In some instances, subsequent individualized optimization was done to improve signals visualization.

## Findings

### DISH optimization protocols

Table 1 summarizes the samples used in this study, and their archived location. Ninety two mEOC samples were evaluated with DISH, of which 79 samples were successfully optimized using a series of optimization protocols developed for this study. Table 2 lists the nominal protocol U1 and four other protocols (U2, U3, U4 and U5) which had parameters modified from U1. All protocol numbers were arbitrarily assigned. In developing the four other protocols (U2, U3, U4 and U5), age of samples was a major consideration during the optimization of parameters from the nominal protocol (U1) for protocols U2, U3, U4 and U5. In general, U1 (nominal protocol) was used for the most recent year 2011 samples on hand, U2 protocol for samples dated between year 2008 and 2010, U3 for samples dated between 2003 and 2007, U4 for samples dated between 2000 and 2002, and U5 for samples dated in the 1990s. Several parameters in the nominal protocol were explored. The following sections describe the reasoning for altering the nominal parameters.

**Table 2:** Nominal and optimization protocols for Dual in-situ hybridization (DISH)

**Selectable parameters**	**U1 (Nominal protocol)**	**U2**	**U3**	**U4**	**U5**
Year of samples	2011	2008-2010	2003-2007	2000-2002	1990s
Baking temperature	63°C	63°C	63°C	63°C	63°C
Baking time	20 mins	20 mins	20 mins	20 mins	20 mins
Deparaffinization	72°C	72°C	72°C	72°C	72°C
Extended deparaffinization	Not selected	Not selected	Not selected	Not selected	Not selected
Cell conditioning duration	3 cycles of CC2 at 86°C	3 cycles of CC2 at 86°C	3 cycles of CC2 at 86°C	3 cycles of CC2 at 86°C	3 cycles of CC2 at 86°C
	Mild CC2: 8 mins	Mild CC2: 8 mins	**Mild CC2: 12 mins**	**Mild CC2: 16 mins**	**Mild CC2: 16 mins**
	Standard CC2: 12 mins	Standard CC2: 12 mins	**Standard CC2: 12 mins**	**Standard CC2: 16 mins**	**Standard CC2: 16 mins**
	Extended CC2: 8 mins	Extended CC2: 8 mins	**Extended CC2: 12 mins**	**Extended CC2: 16 mins**	**Extended CC2: 16 mins**
ISH protease – 2 or 3, and duration	ISH protease 3	**ISH protease 2**	**ISH protease 2**	**ISH protease 2**	**ISH protease2**
	-16 mins	-**8 mins**	-**8 mins**	-**8 mins**	-**12 mins**
Denaturation time	20 mins	20 mins	20 mins	20 mins	20 mins
Hybridization time	6 hours	6 hours	6 hours	6 hours	6 hours
Stringency wash temperature	72°C	72°C	72°C	72°C	**76°C**
SISH multimer incubation time	32 mins	**36 mins**	**36 mins**	**36 mins**	**36 mins**
Silver chromogen incubation time	4 mins	**8 mins**	**8 mins**	**8 mins**	**8 mins**
Red ISH multimer incubation time	24 mins	**28 mins**	**28 mins**	**28 mins**	**28 mins**
Red chromogen incubation time	8 mins	**12 mins**	**12 mins**	**12 mins**	**12 mins**
Hematoxylin II incubation time	8 mins	8 mins	**12 mins**	**12 mins**	**12 mins**
Bluing reagent incubation time	4 mins	4 mins	**8 mins**	**8 mins**	**8 mins**

*CC2*: Cell Conditioning 2. Parameters highlighted in bold indicate changes from nominal protocol U1.

### Cell conditioning 2

With increasing age of samples, stronger pre-treatment of the tissue sections was applied, by increasing the incubation periods of CC2 and/or ISH proteases. The ultra CC2 solution is a citrate buffer at pH 6. It functions to break covalent protein-protein and protein-DNA crosslinks that were formed by formalin in the tissue. The breakage of the bonds is done in conjunction with the heating mechanism on each slide pad. The heat serves to increase the kinetic energy of the molecules that are found in the sample, and the increased motion will cause the bonds that were formed during fixation to break. Removing these bonds would aid in unmasking of the target DNA for hybridization to occur. By increasing the incubation times of the three cycles of CC2 treatment, there is increased probability that the bonds will be broken.

### ISH proteases

Both ISH proteases 2 and 3 serve the same function – they permeabilize cell membranes as the DNA of interest is found intracellularly. It also cleaves peptide bonds of proteins that surround the DNA of interest. The reason for using ISH protease 2 is because it has stronger protease activity in alkaline pH, compared to ISH protease 3.

### Multimers and chromogens

By increasing the incubation time of SISH and Red ISH multimers which are secondary antibodies conjugated with horse radish peroxidase and alkaline phosphatase respectively, there is more time for the secondary antibodies to bind to their respective primary antibodies. Increasing the detection component (i.e. chromogens) incubation times is effective for controlling signal size and/or intensity. This will aid in signal enhancement.

### Hematoxylin II and bluing reagent

The incubation times of Hematoxylin II and bluing reagent were increased in U3, U4 and U5 protocols in the hope that the nuclear morphology would be more easily distinguished by enhancing the blue color within the nucleus. This is because from U3 protocol onwards, there is increased duration of cell conditioning given to the tissue sections. This may cause excessive breakage of the covalent protein-protein and protein-DNA crosslinks in the tissue, therefore causing the nuclear morphology to be less distinguishable.

### Decision workflow for optimization

**Figure 1:**
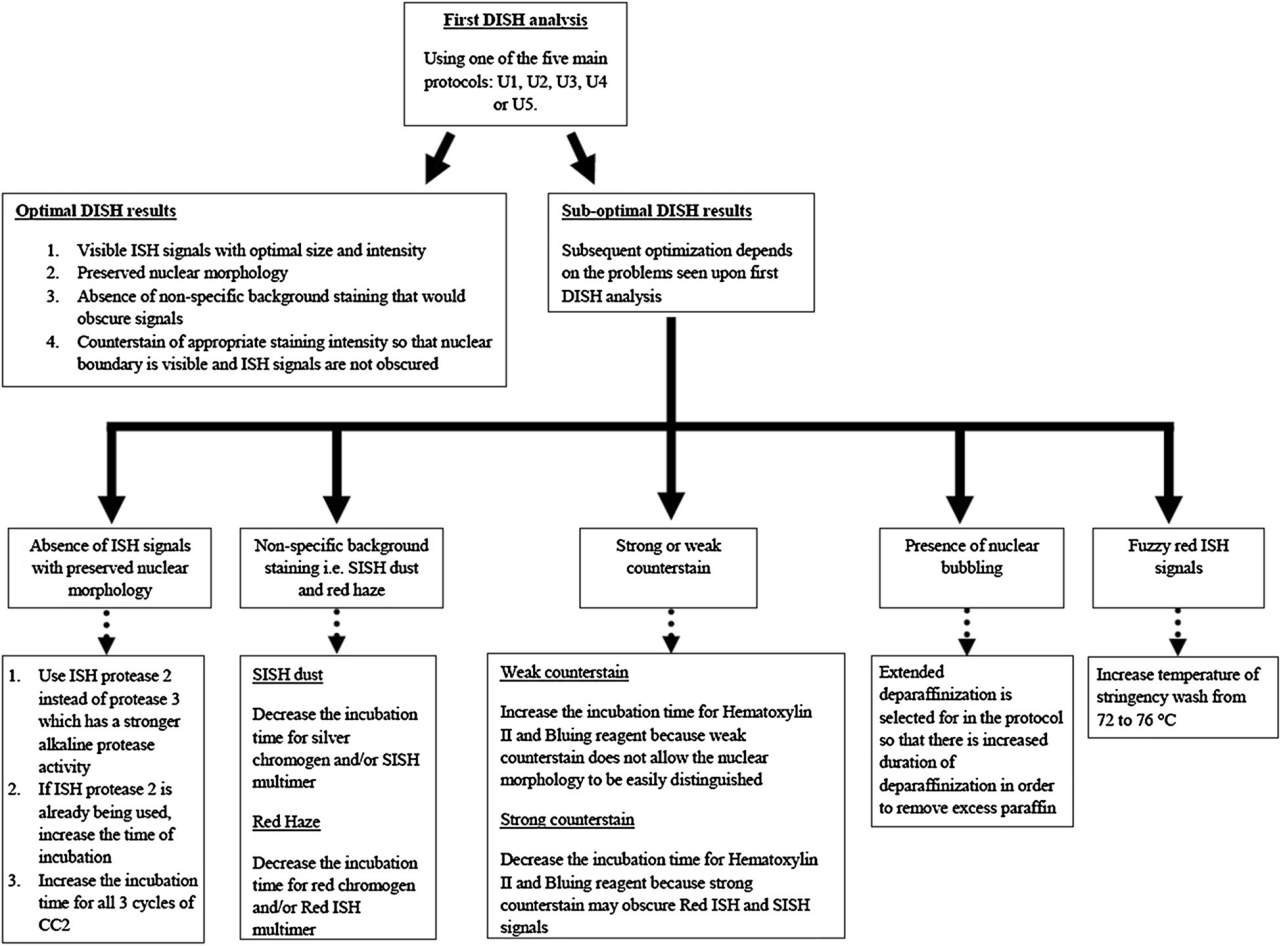
**Decision workflow for optimizing DISH protocols.** Potential problems with corresponding protocols are highlighted in the decision tree.

Figure 1 shows the decision tree that was developed to facilitate the workflow in subsequent individualized optimization. Five main problems were observed upon first DISH optimization (using either of U1, U2, U3, U4 or U5) and highlighted in the workflow. They include: absence of ISH signals with preserved nuclear morphology, presence of non-specific background staining (SISH dust and red haze), strong or weak counterstain, presence of nuclear bubbling, and fuzzy ISH signals. Besides changing the parameters for CC2, ISH proteases, SISH and Red ISH multimers, red and silver chromogens, and Hematoxylin II and bluing reagent according to the problems observed, selection of extended deparaffinization and increasing the temperature of stringency wash were also done in the subsequent individualized optimization, if necessary.

Extended deparaffinization was selected when nuclear bubbling occurred. This happened as a result of excess paraffin in the tissue section. Temperature of stringency wash were increased from 72°C to 76°C if the red signals appeared fuzzy i.e. not a solid and discrete red signal. Incubation duration of counterstains were adjusted for if the nuclear counterstain was too light/dark. Visibility of nuclear boundary might be a problem if the counterstain was light, and obscuration of black signals might occur if the counterstain was too dark. When non-specific background staining like SISH dust and red haze occurred, the incubation times for SISH multimer and silver chromogen, and Red ISH multimer and red chromogen was decreased.

### Individualized optimization

**Figure 2:**
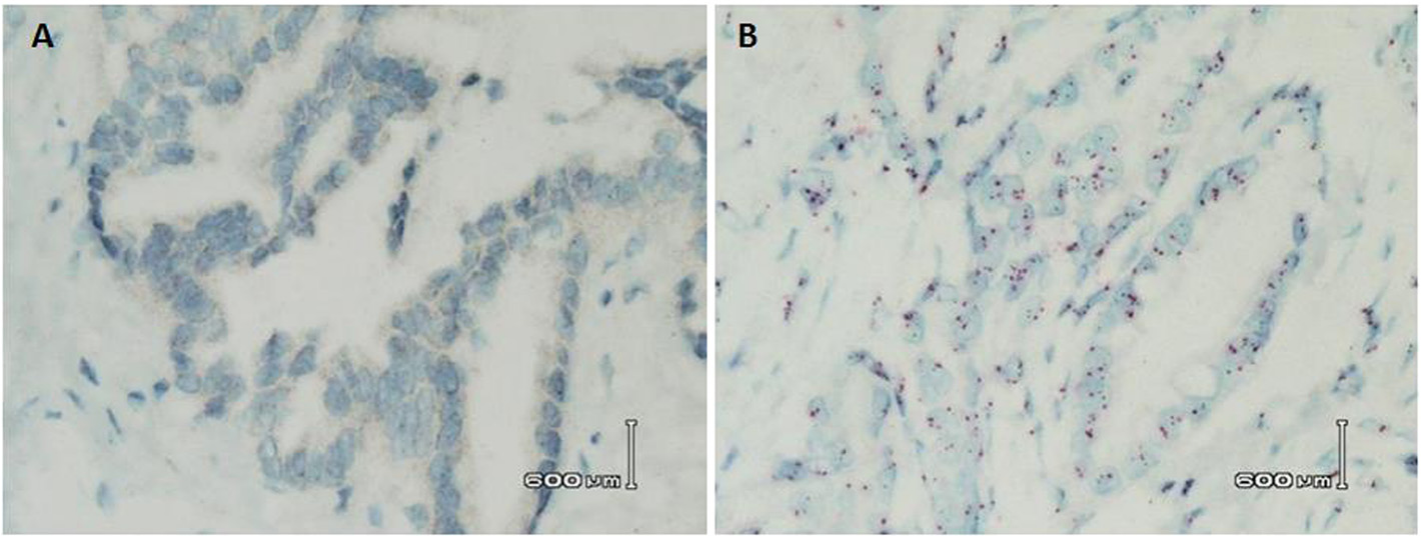
**Results of DISH before and after individualized optimization for a sample from year 2003. (A)** U3 protocol was used for first DISH analysis. There were no red and black signals within the nuclei, and there was SISH dust in the background. As the nuclear morphology was preserved, further optimization could be done to optimize the staining process. **(B)** U6 protocol was used for the second DISH analysis and was successful. There were enumerable red and black signals within the nuclei after a longer duration of cell conditioning with CC2 and longer ISH protease 2 treatments were used. The lengths of incubation for SISH and Red ISH multimers, silver and red ISH chromogens, Hematoxylin II counterstain and bluing reagent were decreased so as to reduce unspecific background staining (SISH dust) and intensity of counterstain. Original magnification **(A, B: 600×)**.

Despite optimization protocols for the samples dated between 1990 and 2010, some samples had to be individually optimized subsequently, depending on the type of problems seen upon first DISH assessment when the respective protocols were applied in accordance to the year of the sample. Figure 2A shows a sample dated year 2003 for which protocol U3 was applied. However, there were no visible red and black signals within the nuclei. A second round of optimization was performed – several parameters in U3 were modified into a new protocol U6. As there were no red and black signals, longer cell conditioning and stronger protease treatment were given, according to the decision workflow in Figure 1. ISH protease 2, which is an alkaline protease of higher strength than ISH protease 3, was already used in the first DISH testing. However, there were no signals. Thus the incubation time for ISH protease 2 was increased from eight minutes to 12 minutes. The duration of three cycles of cell conditioning by CC2 was also increased from 12 minutes to 16 minutes so that there would be sufficient time for CC2 to break covalent protein-protein and protein-DNA crosslinks formed by formalin. This would also aid in unmasking the target DNA. Since there was SISH dust, the incubation periods of SISH multimer and silver chromogen was decreased to 32 and four minutes respectively so as to reduce non-specific background staining. Because increased duration of protease treatment would create more non-specific background staining due to unmasking of more non-specific DNA, the incubation periods of the Red ISH multimer and red chromogen have to be decreased as well, to 24 and eight minutes respectively. As the counterstain was dark with U3 protocol, incubation periods for hematoxylin II and bluing reagent were also decreased to eight and four minutes respectively so that SISH signals will not be obscured. Figure 2B shows the optimal results achieved with subsequent optimization. Table 3 summarized the modified protocol.

**Table 3:** Individualized optimization (U6): protocol used for optimization of a 2003 case and comparison with first DISH protocol U3

**Selectable parameters**	**Protocol used for first DISH analysis (U3)**	**Protocol used for second DISH analysis (U6)**
Baking temperature	63°C	63°C
Baking time	20 mins	20 mins
Deparaffinization	72°C	72°C
Extended deparaffinization	Not selected	Not selected
Cell conditioning duration	3 cycles of cell conditioning 2 (CC2) at 86°C	3 cycles of cell conditioning 2 (CC2) at 86°C
	Mild CC2: 12 mins	Mild CC2: **16 mins**
	Standard CC2: 12 mins	Standard CC2: **16 mins**
	Extended CC2: 12 mins	Extended CC2: **16 mins**
ISH protease – 2 or 3, and duration	ISH protease 2 – 8 mins	ISH protease 2 – **12 mins**
Denaturation time	20 mins	20 minutes
Hybridization time	6 hours	6 hours
Stringency wash temperature	72°C	72°C
SISH multimer incubation time	36 mins	**32 mins**
Silver chromogen incubation time	8 mins	**4 mins**
Red ISH multimer incubation time	28 mins	**24 mins**
Red chromogen incubation time	12 mins	**8 mins**
Hematoxylin II incubation time	12 mins	**8 mins**
Bluing reagent incubation time	8 mins	**4 mins**

The incubation lengths of various selectable parameters that were changed are in bold.

### Unsuccessful optimization

During the first DISH assessment, 13 samples showed inadequately preserved nuclear morphology with no ISH signals throughout the tissue section were not further optimized, and were deemed as unsuccessfully optimized. Optimization was not done for the reason that in order to accentuate the red and black signals, longer cell conditioning and harsher protease treatment had to be given. This would in turn cause poorer nuclear morphology which would affect signal enumeration.

### Effects of age of samples on DISH

**Figure 3:**
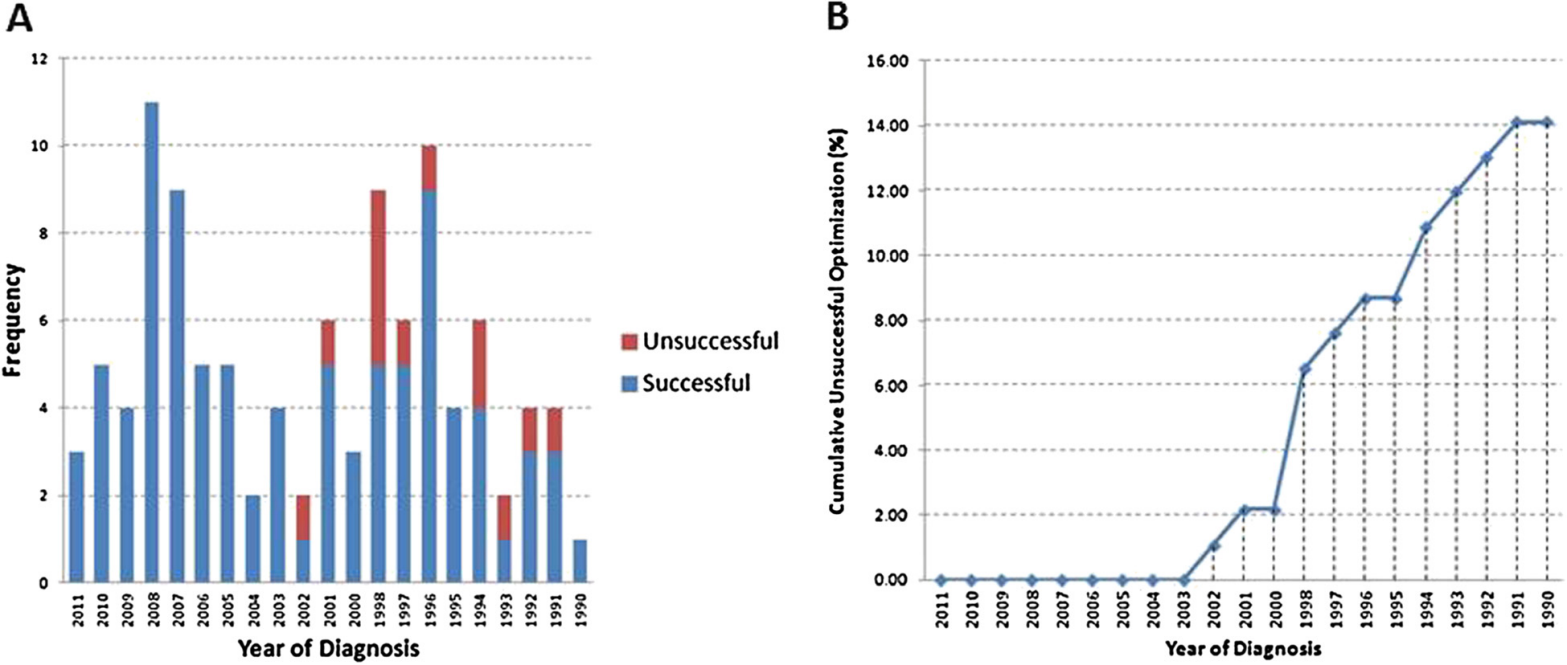
**Effects of age of samples on optimizations. (A)** Number of unsuccessful optimizations from 1990 to 2011. A total of 13 samples were not successfully optimized. All samples dated between 2003 and 2011 were optimized successfully. No unsuccessful optimization was observed in 1990, 1995 and 2000. There was 1 unsuccessful optimization in years 1991, 1992, 1993, 1996, 1997, 2001 and 2002; 2 unsuccessful optimizations in 1994. The most number of samples that were not optimized successfully is from year 1998. **(B)** Cumulative % of samples unsuccessfully optimized.

Age of samples is a factor that was considered during the design of optimization protocols. Samples more than 1 year old needed optimization beyond the nominal protocol. Figure 3A shows the numbers of successful and unsuccessful optimization for the 92 samples. Successful optimizations were achieved for samples dated from year 2003 onwards, regardless of the number of optimizations required. Optimization failures occurred in samples dated before year 2003 which were also those that were archived off-site, as shown in Table 1. Figure 3B shows cumulative percentage of samples with unsuccessful optimization, providing us with a means for deciding future DISH assay based on: (i) age of samples, and (ii) tolerance for unsuccessful optimizations. For example, the plot indicates that for successful DISH experiments (i.e. zero unsuccessful optimization), samples from 2003 onwards (i.e. ≤ 10 years) could be used. These samples were also archived within KKH. Optimization failure happens to samples which were archived off-site and they were also more than 10 years of age. Storage conditions have been shown to be a factor in determining quality of the samples [11-15] which will affect success of DISH assay. In our laboratory archival protocol, samples more than 10 years old in general, were archived off-site. Figure 3A showed samples that were archived off-site, at least 50% from each year could still be optimized successfully. Overall, 76.8% samples archived off-site succeeded in the DISH assay using the optimization protocols. We find Figure 3B useful as a guideline to determine tolerance for failure in optimization, especially when resources are limited. In the case where 5% of samples were allowed to fail optimization, the plot indicates that samples dating from year 2000 onwards could be considered. It should be noted that the analysis shown in Figure 3B is specific to our local context, though the method could be employed in other DISH studies to establish guidelines applicable for their particular environment.

## Discussion

The study explored several parameters in the nominal protocol and formulated a series of optimization protocols and decision workflow for successful DISH assay. Age of samples was a major consideration in the process of developing optimization protocols. Our objective was to improve decision making in choosing of optimization protocols, especially critical when tissue samples are of limited quantity. The workflow in Figure 1 provides a roadmap, covering majority of problems encountered with the assay in older samples. Complications that may arise when staining are non-optimal are: (i) overestimation of the number of red and/or black signals due to non-specific background staining; (ii) overestimation of red and/or black signals due to non-distinct nuclear morphology (bigger nucleus envisioned), or we may underestimate the number of signals (smaller nucleus envisioned). It should be noted the optimization protocols (U2 to U5) was not tested on the more recent fresher samples due to limited amount of tissue samples. In general, we recommend U1 for the fresher samples while U2-U5 would be more applicable for problems observed in older samples.

The study also highlights possible reasons for unsuccessful optimization in older samples, including long archival period and impact of storage conditions of FFPE samples which has been previously reported [11-16]. It showed samples which were archived off-site had a higher proportion of unsuccessful optimizations, as compared to those samples archived at KKH (Table 1). Since age of samples is confounded with off-site storage in this study, we could not tease out individual effects of these two factors. We like to highlight that effects of samples quality tend to be less detrimental on IHC, which was what we observed in this study. For samples that failed DISH, we were able to observe staining using IHC.

Other factors affecting the assay include the temperature at which the FFPE samples were archived at, whether they were protected from the air or sunlight, duration of storage, and tissue fixation [16-19]. The tissue fixation and embedding process, as well as the archival of these FFPE blocks for extended period of time, have a negative influence on the quality of DNA and RNA quality [14]. Storage of FFPE samples at 4°C has been shown to yield the best result; there is minimal fragmentation of the nucleic acid [14]. DNA fragmentation also occurs gradually overtime therefore DNA quality in samples that were archived for longer periods were affected [15]. In addition, exposure to air or sunlight has a negative influence on the quality of the nucleic acid.

Tissue fixation factors such as type of fixative used, cold ischemia time, and fixation time, can also affect optimal staining. At the time of the study, it was not possible to obtain information on the fixation protocols of past years in KKH. However, neutral-buffered formalin (NBF) has been routinely used in KKH over the years. In general, for INFORM HER2 Dual ISH DNA Probe Cocktail Assay to work optimally it was recommended that tissues are fixed in NBF for six to 48 hours; this is consistent with the HER2 testing guidelines established by ASCO/CAP [17]. NBF has been routinely used in KKH but fixation time was not controlled for. The thickness of the tissue will determine the length of time for fixation as fixative penetration into the tissue is a rate-limiting step. Penetration rates decrease with depth. Larger samples would require a longer fixation time, to allow formalin to penetrate the tissue [12]. Diffusion of formalin into the tissue is also taken into consideration for fixation time [11]. Therefore, if insufficient time (< 6 hours) was given for fixation, tissues may be inadequately fixed in the center of the sample. This appeared to be the case for some of the samples where good nuclear morphology with optimal signals and adequate counterstain were observed at the periphery but poor nuclear morphology without any signals in the center. This is because the center of the tissue has undergone tissue autolysis whereby nucleic acids are degraded by the endogenous nucleases [14]. In order to reduce tissue autolysis, one recommendation is to keep the thickness of the sample at 5 mm so as to ensure an even fixation throughout the sample. Samples that undergo autolysis have a loss in hybridization capability [12]. Unsuccessful optimization could also be due to over-fixation of tissues (>48 hours) which causes an increased number of irreversible crosslinks [11,14]. The time from which the sample is removed from the patient to the time of fixation which is also known as cold ischemia time, is also crucial to successful DISH analysis. Ideally, it should be < 1 hour, according to ASCO/CAP guidelines [18]. The time to fixation is of utmost importance because cellular processes and tissue autolysis can take place during this time interval [14]. If the time between sample collection and fixation is shortened, DNA quality will be improved [12]. Therefore, it is important that after collection, samples are sent immediately to the histopathology laboratory for processing [12].

DISH assay has been introduced as an alternative to FISH in the evaluation of HER2 gene amplification. Although FISH has been the gold standard for HER2 evaluation in cancers, it has several limitations. This includes inability to visualize nuclear morphology for differentiation of tumor tissue and normal tissue during signal enumeration; fading of fluorescent signals over time resulting in the inability to archive, and high cost [1,17,19]. It also requires expertise and fluorescence imaging system for interpretation [1,19]. Both FISH and DISH techniques are based on the principle of hybridization of probes to their target sequences, i.e. the HER2 gene as well as the alpha satellite repeats in the centromere of chromosome 17 [20]. However, DISH and FISH differ in their detection due to the different probe labeling method. In FISH, the probes are labeled with fluorophores and thus detected fluorescently. In DISH, the probes are labeled with haptens which can be detected under light microscope. DISH is a combination of silver in-situ hybridization (SISH) and red in-situ hybridization (Red ISH), and it enables visible nuclear morphology allowing differentiation of normal and tumor tissue in conjunction with HER2 gene amplification status. Interpretation is based on light microscopy which is more user-friendly and the slides are archivable.

## Conclusion

This study developed an optimization decision workflow and a series of optimization protocols for DISH assay. It highlighted the potential problems that can be encountered in DISH and recommends alterations in six primary parameters: CC2, ISH protease, temperature of stringency wash, SISH and ISH multimers, silver and red chromogens, and Hematoxylin II and bluing reagent to improve success of the assay. In instances when the first round of optimization was unsuccessful (i.e. no visible signals with preserved nuclear morphology), stronger pretreatment were given to enhance signals. Also, if other problems surfaced during the first round of optimization, parameters were altered consequently. A decision workflow has been developed to provide guidelines on this. Overall, the major factors affecting the success of DISH are age of samples and storage conditions. Samples more than 10 years and archived off-site tend to show more failures in optimization.

## Abbreviations

AP: Alkaline phosphatase; CC2: Cell Conditioning 2; DIG: Digoxigenin; DISH: Dual in-situ hybridization; DNP: Dinitrophenyl; FFPE: Formalin-fixed paraffin-embedded; FISH: Fluorescence in-situ hybridization; H&E: Hematoxylin and eosin; HER2: Human epidermal growth factor receptor 2; HRP: Horse radish peroxidase; KKH: Kandang Kerbau Women’s and Children’s Hospital; LCS: Liquid coverslip; mEOC: Mucinous epithelial ovarian cancer; SSC: Sodium citrate sodium chloride.

## Competing interests

The authors declare that they have no competing interests.

## Authors’ contributions

XYL: Carried out the experiments, drafted and revised the manuscript. CSH: Participated in DISH and immunoassays, and interpretation of data. LTSK: Participated in DISH and immunoassays. CWY: Contributed to the patient cohort. GLK: Design of study, interpretation of data, drafting and revision of manuscript. All authors read and approved the final manuscript.
